# Publisher Correction: A space–time tradeoff for implementing a function with master equation dynamics

**DOI:** 10.1038/s41467-019-10162-8

**Published:** 2019-05-01

**Authors:** David H. Wolpert, Artemy Kolchinsky, Jeremy A. Owen

**Affiliations:** 1 Santa Fe Institute, 1399 Hyde Park Road, Santa Fe, NM 87501 USA; 2 Arizona State University, Tempe, 85281 AZ USA; 3 Physics of Living Systems Group, Department of Physics, Massachusetts Institute of Technology, 400 Tech Square, Cambridge, MA 02139 USA

**Keywords:** Information theory and computation, Statistical physics, thermodynamics and nonlinear dynamics

Correction to: *Nature Communications* 10.1038/s41467-019-09542-x, published online 15 April 2019.

The original version of the Supplementary Information associated with this Article contained an error throughout, in which all inline references to Theorems, Definitions, and Lemmas given in the main Article were incorrectly given as ‘??’. The HTML has been updated to include a corrected version of the Supplementary Information.

