# Supplementary material for: A space–time tradeoff for implementing a function with master equation dynamics
Source: Nat Commun. 2019 Apr 15;10:1727. doi: 10.1038/s41467-019-09542-x (PMC6465315; doi:10.1038/s41467-019-09542-x)
Supplement: Supplementary file 1 — Supplementary Information [file 41467_2019_9542_MOESM1_ESM.pdf]

Supplementary Information

A space-time tradeoff for implementing a function with master equation dynamics

Wolpert et al.

Supplementary Note 1: Explicit demonstration that bit erasure is a one-step function

In the model of bit erasure described in [1] a classical bit is stored in a quantum dot, which can be either empty (state 0) or filled with an electron (state 1). The dot is brought into contact with a metallic lead at temperature T which can transfer an electron to/from the dot. The propensity of the lead to give an electron is set by its chemical potential, indicated by $\mu(t)$ at time t. The energy of an electron in the dot is indicated by $E(t)$.

Let $p(t)$ indicate the two-dimensional vector of probabilities at time t, with $p_0(t)$ and $p_1(t)$ being the probability of an empty and full dot, respectively. These probabilities evolve according to a rate matrix [1]:

$$\dot{p}(t) = C \begin{bmatrix} -w(t) & 1 - w(t) \\ w(t) & -(1 - w(t)) \end{bmatrix} p(t) \tag{1}$$

where C sets the timescale of the exchange of electrons between the dot and the lead and $w(t)$ is the Fermi distribution of the lead,

$$w(t) = \left[\exp((E(t) - \mu(t))/k_B \mathrm{T}) + 1\right]^{-1} . \tag{2}$$

Using Eq. (1) and conservation of probability (i.e., $p_0(t) + p_1(t) = 1$), we can write

$$\dot{p_1}(t) = C(w(t) - p_1(t)) , \tag{3}$$

so $p_1(t) = w(t)$ is the stationary state at time t.

Suppose that the chemical potential $\mu(t)$ and electron energy $E(t)$ are chosen in such a way that $w(t) = (1 - t)q + t\delta$ for some constants q and δ. In this case, Eq. (3) can be explicitly solved for p_1,

$$p_1(t) = w(t) + e^{-Ct} (p_1(0) - q) + C^{-1}(q - \delta) \left(1 - e^{-Ct}\right) . \tag{4}$$

In the limit where $C \to \infty$ and $\delta \to 0$, we have

$$p_1(t) = w(t) = (1 - t)q , \tag{5}$$

which corresponds to the transition matrix

$$T(0, t) = \begin{bmatrix} 1 - (1 - t)q & 1 - (1 - t)q \\ (1 - t)q & (1 - t)q \end{bmatrix} . \tag{6}$$

Note that $T(0,1) = \left(\begin{smallmatrix} 1 & 1 \\ 0 & 0 \end{smallmatrix}\right)$, so the process implements bit erasure. By Lemma 16, it must also be that $T(t,1) = \left(\begin{smallmatrix} 1 & 1 \\ 0 & 0 \end{smallmatrix}\right)$. We note that $T(0,t)$ and $T(t,1)$ are continuous in t and have a constant set of allowed transitions over $t \in (0,1)$, which establishes that bit erasure is one-step.

Supplementary Note 2: Properties of master equations that implement idempotent functions in one timestep

We begin by proving that any idempotent function over a finite $\mathcal{X}$ is one-step, Theorem 4. Let f be an idempotent function, and let $P_{ij} = \delta(i, f(j))$ be the corresponding stochastic matrix. We use an explicit construction to show that there exists a sequence of CTMCs $\{T^{(n)} : n = 1, 2, \ldots\}$ which obey the conditions of Definition 6.

First, choose any arbitrary probability distribution q over $\mathcal{X}$, and let q_i indicate the probability of state i. Define

$$\tilde{q}_i := \begin{cases} q_i / \sum_{j:f(j)=f(i)} q_j & \text{if } \sum_{j:f(j)=f(i)} q_j > 0 \\ 0 & \text{otherwise} \end{cases} \tag{1}$$

Each $\tilde{q}_i$ is the 'renormalized' probability within the block of states $\{j : f(j) = f(i)\}$.

Then, for all i, define

$$w_i(t) = (1-t)\tilde{q}_i + t\delta(i, f(i)) \,. \tag{2}$$

where $\delta(\cdot, \cdot)$ is the Kronecker delta function.

Then, define the rate matrix $Q^{(n)}(t)$ as

$$Q^{(n)}_{ij}(t) = \begin{cases} n w_i(t) & \text{if } i \neq j \text{ and } f(i) = f(j) \\ n(w_i(t) - 1) & \text{if } i = j \\ 0 & \text{otherwise} \end{cases} \tag{3}$$

It can be verified that if f is an idempotent function, then $Q^{(n)}(t)$ is a valid rate matrix (that is, $Q^{(n)}_{ij}(t) \geq 0$ for all i, j and $\sum_i Q^{(n)}_{ij}(t) = 0$ for all j).

Next, for any $n \in \mathbb{N}$, define the CTMC $T^{(n)}(t, t')$ as the solution to the following differ-

ential equation,

$$T_{ij}^{(n)}(t,t) = \delta(i,j) \tag{4}$$

$$\frac{d}{dt'} T_{ij}^{(n)}(t,t') = \sum_k Q_{ik}^{(n)}(t') T_{kj}^{(n)}(t,t') \tag{5}$$

We can simplify Eq. (5) by using the definition of $Q^{(n)}(t)$. First note that no probability can ever flow from state j to state i if $f(i) \neq f(j)$, hence for such i,j, $T_{ij}^{(n)}(t,t') = 0$ always. On the other hand, for i,j where $f(i) = f(j)$, we can rewrite

$$\begin{aligned}
\frac{d}{dt'} T_{ij}^{(n)}(t,t') &= \sum_k Q_{ik}^{(n)}(t') T_{kj}^{(n)}(t,t') \\
&= n \left[(w_i(t') - 1) T_{ij}^{(n)}(t,t') + \sum_{k: k \neq i, f(k) = f(i)} w_i(t') T_{kj}^{(n)}(t,t') \right] \\
&= n \left((w_i(t') - 1) T_{ij}^{(n)}(t,t') + w_i(t') \left(1 - T_{ij}^{(n)}(t,t') \right) \right) \\
&= n \left(w_i(t') - T_{ij}^{(n)}(t,t') \right)
\end{aligned} \tag{6}$$

Eq. (6), in combination with initial condition Eq. (4), can be explicitly solved to give

$$T_{ij}^{(n)}(t,t') = w_i(t') + (\delta(i,j) - w_i(t)) e^{-(t'-t)/n} + n^{-1} (\tilde{q}_i - \delta(i, f(i)))(1 - e^{-(t'-t)/n}) \,. \tag{7}$$

The $n \to \infty$ limit for $t' \geq t$ is

$$T_{ij}(t,t') := \lim_{n \to \infty} T^{(n)}(t,t') = \begin{cases} w_i(t') & \text{if } t' > t \text{ and } f(i) = f(j) \\ 0 & \text{if } t' > t \text{ and } f(i) \neq f(j) \\ \delta(i,j) & \text{if } t = t' \end{cases} \tag{8}$$

As a particular case, for $t = 0, t' = 1$, we have

$$T_{ij}(0,1) = \delta(i, f(j)) = P \,, \tag{9}$$

where we've used the fact that $w_i(1) = \delta(i, f(i))$.

We have thus shown that $\{T^{(n)} : n = 1, 2, \dots\}$ is a limit-embedding of P, as required for any one-step matrix. Next, the condition Definition 6(1) on the sequence $\{T^{(n)} : n = 1, 2, \dots\}$ is met by inspection. In addition, since $w_i(t)$ is a continuous function of $t \in [0,1]$, it follows both that $T(0,t)$ is a continuous function of t for all $t \in (0,1]$ and that $T(t,1)$ is a continuous function of t for all $t \in [0,1)$. This establishes that Definition 6(2) holds. Finally, Definition 6(3) holds by construction.

Thus, all the conditions given in Definition 6 concerning the limiting matrix $T(t, t')$ are satisfied, which establishes the claim that P is a one-step matrix. Note in particular that even though $\{T^{(n)} : n = 1, 2, \dots\}$ is defined in terms of one particular initial distribution q, the associated transition matrix $T(0, t)$ implements P no matter what the initial distribution is.

It is worth highlighting three properties of the construction above.

First, when q equals the initial distribution $p(0)$, the function $p(t) = T(0, t)p(0)$ is a continuous function of t for all $t \in [0, 1]$. To see this, first note that since $T(0, t)$ is continuous for all $t \in (0, 1]$, $T(0, t)p(0)$ is continuous for all $t \in (0, 1]$. Moreover,

$$\begin{aligned}
\lim_{t\to 0^+} p_i(t) &= \lim_{t\to 0^+} \sum_j T_{ij}(0, t) p_j(0) \\
&= \sum_{j:f(j)=f(i)} w_i(0) p_j(0) \\
&= \sum_{j:f(j)=f(i)} \frac{q_i}{\sum_{j':f(j')=f(i)} q_j} p_j(0) = p_i(0)
\end{aligned} \tag{10}$$

Therefore $p(t)$ in fact is continuous for all $t \in [0, 1]$, as claimed.

Second, when $q = p(0)$, then the above construction results in no (irreversible) entropy production. More precisely, stochastic thermodynamics provides a simple formula for the rate of entropy production incurred by a system evolving according to a master equation, while being coupled to a thermodynamic reservoir [2, 3]:

Proposition 15*:* Consider a CTMC with finite rates $Q(t)$ and let $p(t)$ be a distribution of states at time t of a system that evolves according to that CTMC. The (irreversible) entropy production rate at time t is

$$\dot{\Sigma}(Q(t), p(t)) := \sum_{i,j} p_j(t) Q_{ij}(t) \ln \frac{p_j(t) Q_{ij}(t)}{p_i(t) Q_{ji}(t)} \tag{11}$$

The integrated entropy production over $t \in [0, 1]$ is

$$\Sigma(Q, p(0)) = \int_0^1 \dot{\Sigma}(Q(t), p(t))\, dt\,. \tag{12}$$

Now consider the rate matrices $Q^{(n)}(t)$ defined in Eq. (3). Note that for all $t \in [0, 1]$, these rate matrices have a fixed "block structure", in which transitions are allowed between states i, j in the same block ($f(i) = f(j)$), but not allowed between states i, j in different blocks ($f(i) \neq f(j)$). It is straightforward to verify that for block-structure rate matrices, one can

rewrite Eqs. (11) and (12) as a weighted sum of entropy production terms arising from each block. In particular, letting $S_k = f^{-1}(k)$ be the preimage of k under f, we can rewrite Eq. (12) as

$$\Sigma(Q^{(n)}, p(0)) = \sum_k p^k(0)\, \Sigma\left(Q^{(n)}_{[S_k]}, p_{[S_k]}(0)/p^k(0)\right)\,, \tag{13}$$

where $p^k(0) = \sum_{i \in S_k} p_i(0)$, $p_{[S_k]}$ is the restriction of the distribution p to the states in S_k, and $Q^{(n)}_{[S_k]}$ uses the notation from Definition 3. Then, each $Q^{(n)}_{[S_k]}$ is irreducible and (if $q = p(0)$) exactly follows the construction specified in the Appendix D of the companion paper [4]. In that Appendix, we prove that

$$\lim_{n\to\infty} \Sigma\left(Q^{(n)}_{[S_k]}, p_{[S_k]}(0)/p^k(0)\right) = 0\,. \tag{14}$$

Thus, in the $n \to \infty$ limit, the integrated entropy production vanishes.

Third, we note that we can build a CTMC that implements a composition of idempotents by "gluing together" the CTMC corresponding to each idempotent in turn. For example, suppose we wish to implement a map $h = f \circ g$, where f and g are idempotents with corresponding stochastic matrices P_1, P_2. Write $Q_1^{(n)}$ and $Q_2^{(n)}$ for the rate matrices implementing P_1 and P_2 respectively (as in Eq. (3)). Then, we can implement h by taking the $n \to \infty$ limit of the rate matrices

$$Q^{(n)}(t) = \begin{cases} Q_1^{(n)}(2t) & \text{if } t \in [0, \frac{1}{2}] \\ Q_2^{(n)}(2t-1) & \text{if } t \in (\frac{1}{2}, 1] \end{cases}\,. \tag{15}$$

Supplementary Note 3: Transitivity condition on one-step matrices

One particularly useful property of one-step matrices involves a kind of transitivity of probability flow, formalized as follows:

Definition 10*:* A stochastic matrix P is transitive if for all triples of states $\{i, j, k\}$ such that $P_{ji} > 0$ and $P_{kj} > 0$, it is also true that $P_{ki} > 0$.

In this Supplementary Note we show that one-step matrices are transitive. To do this we start with a pair of simple lemmas. In all of them we take P to be a matrix that is limit-embeddable by T, and such that the limit $T(t,t') := \lim_{n\to\infty} T^{(n)}(t,t')$ exists for all $t, t' \in [0,1]$.

Lemma 16*:* For any $t \in [0,1]$, $T(0,1) = T(t,1)T(0,t)$.

Proof. Note that any embeddable CTMC in the sequence $T^{(n)}$ obeys the Chapman-Kolmogorov equations,

$$T^{(n)}(0,1) = T^{(n)}(t,1)T^{(n)}(0,t) \tag{1}$$

Since the limit of a product is the product of limits, we can write

$$\begin{aligned} T(0,1) &= \lim_{n\to\infty} T^{(n)}(0,1) \\ &= \lim_{n\to\infty} T^{(n)}(t,1)T^{(n)}(0,t) \\ &= \left(\lim_{n\to\infty} T^{(n)}(t,1)\right)\left(\lim_{n\to\infty} T^{(n)}(0,t)\right) \\ &= T(t,1)T(0,t)\,. \end{aligned} \tag{2}$$

Lemma 17*:* If P is one-step and $T_{ij}(0,1) > 0$ for some pair of states i and j, then $T_{ij}(0,t) > 0$ for all $t \in (0,1)$.

Proof. If $T_{ij}(0,1) > 0$, by continuity of $T(0,t)$ in t, there must be a $t' \in (0,1)$ such that $T_{ij}(0,t') > 0$. The claim follows from the definition of a one-step matrix.

Theorem 18*:* If P is one-step it is transitive.

Proof. Recall that $P = T(0,1)$, and consider any three states i, j, and k such that $T_{ji}(0,1) > 0$ and $T_{kj}(0,1) > 0$. Given that $T_{kj}(0,1) > 0$, by continuity of $T(t,1)$ in t there must be a $t' \in (0,1)$ such that $T_{kj}(t',1) > 0$. By Lemma 17, given that $T_{ji}(0,1) > 0$, $T_{ji}(0,t) > 0$ for all $t \in (0,1)$. Combining with Lemma 16 gives

$$T_{ki}(0,1) = \sum_{j'} T_{kj'}(t',1)T_{j'i}(0,t') \geq T_{kj}(t',1)T_{ji}(0,t') > 0\,. \tag{3}$$

Thus, if $P_{ji} > 0$ and $P_{kj} > 0$, $P_{ki} > 0$.

Supplementary Note 4: Calculating time cost using products of idempotent functions

For convenience, in this Supplementary Note we define the adjacency matrix of a matrix K as

$$\mathscr{A}[K]_{ij} = \begin{cases} 1 & \text{if } K_{ij} > 0 \\ 0 & \text{otherwise} \end{cases}\,. \tag{1}$$

It can be verified that condition 3 of Definition 6 (one-step matrix) is equivalent to stating that $\mathscr{A}[T(0,t)]$ is constant over $t \in (0,1)$.

We also use $\mathscr{A}[\tilde{L}]_{ij} \subseteq \mathscr{A}[L]_{ij}$ to indicate that $\mathscr{A}[\tilde{L}]_{ij} = 0$ whenever $\mathscr{A}[L]_{ij} = 0$, for all states i, j.

Lemma 19*:* For any one-step matrix L, there exists a one-step matrix $\tilde{L}$ which carries out an idempotent function and which has $\mathscr{A}[\tilde{L}] \subseteq \mathscr{A}[L]$.

Proof. Let G be the graph that corresponds to $\mathscr{A}[L]$. Since L is a stochastic matrix, every node in G must have at least one outgoing edge. Since the number of nodes is finite, this means that there must be a path from every node to at least one node in a directed cycle. Furthermore, since L is one-step, G must be transitive (Theorem 18). Thus, every node must have at least one direct edge to a node in a cycle. Furthermore, for any node in a cycle, there is a directed path from itself back to itself. Since G is transitive, any node in a cycle must therefore have an edge to itself (self-loop).

Thus, any node in G must either have a self-loop, or must be directly connected to at least one other node with a self-loop. For each node without a self-loop, let v_i indicate any node that i is connected to and which has a self-loop. Define the stochastic matrix $\tilde{L}$ in the following manner: for any node i and all j, let $\tilde{L}_{ji} = \delta_{i,j}$ if i has a self-loop, and let $\tilde{L}_{ji} = \delta_{j,v_i}$ if i doesn't have a self-loop. By construction, $\mathscr{A}[\tilde{L}] \subseteq \mathscr{A}[L]$. It is straightforward to check that $\tilde{L}$ is idempotent: every i with a self-loop is sent to itself no matter how many times $\tilde{L}$ is applied, and every i without a self-loop is sent to v_i, no matter how many times $\tilde{L}$ is applied.

$\tilde{L}$ is one-step by Theorem 4.

Lemma 20*:* Consider two stochastic matrices A and $\tilde{A}$ over $\mathcal{Y}$, each expressible as a product of n stochastic matrices,

$$A = L^{(n)} L^{(n-1)} \dots L^{(1)} \qquad\qquad \tilde{A} = \tilde{L}^{(n)} \tilde{L}^{(n-1)} \dots \tilde{L}^{(1)} \tag{2}$$

If for all $i = 1..n$, $\mathscr{A}[\tilde{L}^{(i)}] \subseteq \mathscr{A}[L^{(i)}]$, then $\mathscr{A}[\tilde{A}] \subseteq \mathscr{A}[A]$.

Proof. Define the following partial products,

$$A^{[k]} = L^{(k)} L^{(k-1)} \dots L^{(1)} \qquad\qquad \tilde{A}^{[k]} = \tilde{L}^{(k)} \tilde{L}^{(k-1)} \dots \tilde{L}^{(1)} \tag{3}$$

We prove the Lemma, i.e., that $\mathscr{A}[\tilde{A}^{[n]}] \subseteq \mathscr{A}[A^{[n]}]$, by induction in k.

Observe that since $A^{[1]} = L^{(1)}$ and $\tilde{A}^{[1]} = \tilde{L}^{(1)}$, by assumption $\mathscr{A}[\tilde{A}^{[1]}] \subseteq \mathscr{A}[A^{[1]}]$. Now write

$$A_{ij}^{[k]} = \sum_l L_{il}^{(k)} A_{lj}^{[k-1]} \tag{4}$$

If $A_{ij}^{[k]} = 0$, this means that $\forall l \in \mathcal{Y}$, $L_{il}^{(k)} = 0$ and $A_{lj}^{[k-1]} = 0$. But since $\mathscr{A}[\tilde{L}^{(k)}] \subseteq \mathscr{A}[L^{(k)}]$, $L_{il}^{(k)} = 0$ implies $\tilde{L}_{il}^{(k)} = 0$; similarly, given $\mathscr{A}[\tilde{A}_{lj}^{[k-1]}] \subseteq \mathscr{A}[A_{lj}^{[k-1]}]$, $A_{lj}^{[k-1]} = 0$ implies $\tilde{A}_{lj}^{[k-1]} = 0$. Thus, if $A_{ij}^{[k]} = 0$, then it must be that

$$\tilde{A}_{ij}^{[k]} = \sum_l \tilde{L}_{il}^{(k)} \tilde{A}_{lj}^{[k-1]} = 0 \tag{5}$$

Therefore, if $\mathscr{A}[\tilde{A}_{lj}^{[k-1]}] \subseteq \mathscr{A}[A_{lj}^{[k-1]}]$ and $\mathscr{A}[\tilde{L}^{(k)}] \subseteq \mathscr{A}[L^{(k)}]$, then $\mathscr{A}[\tilde{A}_{lj}^{[k]}] \subseteq \mathscr{A}[A_{lj}^{[k]}]$.

Lemma 5*:* Suppose the stochastic matrix P over $\mathcal{Y} \supseteq \mathcal{X}$ has time cost ℓ and the restriction of P to $\mathcal{X}$ is a function $f : \mathcal{X} \to \mathcal{X}$. Then there is a product of ℓ idempotent functions over $\mathcal{X}$ whose restriction to $\mathcal{X}$ equals f.

Proof. By hypothesis we can write $P = L^{(\ell)} L^{(\ell-1)} \ldots L^{(1)}$ where each $L^{(i)}$ is one-step. By Lemma 19, for each $L^{(i)}$ there is another one-step matrix $\tilde{L}^{(i)}$ which carries out an idempotent function, and which has $\mathscr{A}[\tilde{L}^{(i)}] \subseteq \mathscr{A}[L^{(i)}]$. By Lemma 20, the product of these idempotent functions, $\tilde{P} = \tilde{L}^{(\ell)} \tilde{L}^{(\ell-1)} \ldots \tilde{L}^{(1)}$, obeys $\mathscr{A}[\tilde{P}] \subseteq \mathscr{A}[P]$.

The restriction of P to $\mathcal{X}$ implements the single-valued function $f : \mathcal{X} \to \mathcal{X}$, meaning that $P_{ji} = \delta_{f(i),j}$ for all $i \in \mathcal{X}$. Therefore, it must be that $\tilde{P}_{ji} = \delta_{f(i),j}$ for all $i \in \mathcal{X}$, since otherwise $\tilde{P}$ would have a nonzero entry in a location where P has a 0 entry (contradicting $\mathscr{A}[\tilde{P}] \subseteq \mathscr{A}[P]$). Therefore, the restriction of $\tilde{P}$ to $\mathcal{X}$ must equal f.

As an aside, Lemma 5 tells us that if $\mathcal{X} = \mathcal{Y}$, and P is single-valued and one-step (so $\ell = 1$), then P must be an idempotent function.

Supplementary Note 5: Time cost where visible states are macrostates

Theorem 13*:* Assume $\hat{f} : \mathcal{Z} \to \mathcal{Z}$ can be implemented with n microstates and ℓ timesteps. Then there is a stochastic matrix W over a set of n states $\mathcal{Y}$, a subset $\mathcal{X} \subseteq \mathcal{Y}$ with $|\mathcal{X}| = |\mathcal{Z}|$, and a one-to-one mapping $\omega : \mathcal{Z} \to \mathcal{X}$ such that

1. W is a product of ℓ one-step matrices

2. The restriction of W to $\mathcal{X}$ carries out the function $f(x) := \omega(\hat{f}(\omega^{-1}(x))$

Proof. Assume $\hat{f}$ is implemented with n microstates and ℓ timesteps by the coarse-graining function g and stochastic matrix M. By definition, $M = L^{(\ell)}L^{(\ell-1)}\dots L^{(1)}$ where each $L^{(i)}$ is one-step. By Lemma 19 and Lemma 20, there exists a matrix

$$V = \tilde{L}^{(\ell)}\tilde{L}^{(\ell-1)}\dots\tilde{L}^{(1)} \tag{1}$$

which obeys $\mathscr{A}[V] \subseteq \mathscr{A}[M]$, and where each $\tilde{L}^{(i)}$ carries out an idempotent function.

Condition 3 of Definition 8 states that $\sum_{j\in g^{-1}(\hat{f}(g(i)))} M_{ji} = 1$ for all $i \in \mathrm{dom}(g)$, or equivalently that $\sum_{j\notin g^{-1}(\hat{f}(g(i)))} M_{ji} = 0$. Since the set of zero entries in V is a superset of those in M, it is easy to see that if M satisfies Condition 3, then so must V. Thus, g and V also implement $\hat{f}$ with n microstates and ℓ timesteps.

Let $\gamma : \mathcal{Y} \to \mathcal{Y}$ represent the idempotent function carried out by $\tilde{L}^{(\ell)}$. Define the set

$$D := \mathrm{img}(\gamma) \cap \mathrm{dom}(g)\,, \tag{2}$$

so that D is the set of microstates which are in the image of γ and which have a macrostate defined. Note that the image of any idempotent function consists only of fixed points of that function. Since $D \subseteq \mathrm{img}(\gamma)$, D thus contains only fixed points of γ.

We now define a one-to-one function $\omega : \mathcal{Z} \to \mathcal{Y}$ from macrostates to microstates which maps every macrostate z to one particular "canonical" microstate contained in that macrostate. Formally, we require ω to obey the following two conditions (any ω which obeys these conditions suffices):

1. For all $z \in g(D)$, $\omega(z) \in g^{-1}(z) \cap D$ (i.e., every macrostate that has a microstate in D is mapped by ω to one of its own microstates in D)
2. For all $z \notin g(D)$, $\omega(z) \in g^{-1}(z)$ (i.e., every microstate that does not have a microstate in D is mapped to one of its own microstates)

Note that ω is one-to-one since the sets $g^{-1}(z)$ are non-overlapping for different z. Note also that for any $y \in \mathrm{img}(\omega)$, $\omega^{-1}(y) = g(y)$.

We now construct a "modified" function $\gamma' : \mathcal{Y} \to \mathcal{Y}$ in the following manner,

$$\gamma'(y) = \begin{cases} \omega(g(\gamma(y))) & \text{if } \gamma(y) \in D \\ \gamma(y) & \text{otherwise} \end{cases} \tag{3}$$

In words, γ' is similar to γ, but its outputs are canonical microstates where possible. Below, we show two things: first that γ' is idempotent, and second that if we replace γ by γ', we will still implement $\hat{f}$.

To show that γ' is idempotent, we demonstrate that $\mathrm{img}(\gamma')$ consists only of fixed points of γ'. To do so, we consider two cases separately:

1. $y \in \mathcal{Y}$ with $\gamma(y) \notin D$, for which $\gamma'(y) = \gamma(y)$. Note that since $\gamma(y)$ is idempotent, $\gamma(\gamma(y)) = \gamma(y) \notin D$, and therefore $\gamma'(\gamma'(y)) = \gamma'(\gamma(y)) = \gamma(\gamma(y)) = \gamma(y) = \gamma'(y)$.
2. $y \in \mathcal{Y}$ with $\gamma(y) \in D$, for which $\gamma'(y) = \omega(g(\gamma(y))$. In this case, $g(\gamma(y)) \in g(D)$, so by construction $\omega(g(\gamma(y))) \in D$, thus $\gamma'(\gamma'(y)) = \gamma'(\omega(g(\gamma(y)))) = \omega(g(\gamma(\omega(g(\gamma(y))))))$. As mentioned above, all elements in D are fixed points of γ, so we can write $\gamma(\omega(g(\gamma(y)))) = \omega(g(\gamma(y)))$ to give $\gamma'(\gamma'(y)) = \omega(g(\omega(g(\gamma(y)))))$. Furthermore, by construction of ω, $\omega(z) \in g^{-1}(z)$, thus $g(\omega(g(\cdot)) = g(\cdot)$, so we can further rewrite $\gamma'(\gamma'(y)) = \omega(g(\gamma(y)) = \gamma'(y)$.

This proves that γ' is idempotent.

We now show that we still implement $\hat{f}$ if instead of the last matrix carrying out γ, it instead carries out γ'. Let $\tilde{L}'^{(\ell)}$ be the one-step matrix that encodes function γ', and define the stochastic matrix

$$W = \tilde{L}'^{(\ell)} \tilde{L}^{(\ell-1)} \dots \tilde{L}^{(1)} \tag{4}$$

Now consider any $i \in \mathcal{Y}$, and let j indicate the output state such that $V_{ji} = 1$, where V is as defined in Eq. (1). Let $j' \in \mathcal{Y}$ be the final state such that $W_{j',i} = 1$. We now note two things:

(a) By the definition of γ' in Eq. (3), it must be that either $j' = j$ (in case $j \notin D$) or $j' = \omega(g(j))$ (in case $j \in D$). In either case, $g(j') = g(j)$ (in the former case trivially, and in the latter case since $g(\omega(g(\cdot)) = g(\cdot)$, as mentioned before). It is easy to verify that if Condition 3 of Definition 8 holds for V, it must also hold for W; thus, W in Eq. (4) also implements $\hat{f}$ with n microstates and ℓ timesteps.

(b) Consider the case when $i \in \mathrm{dom}(g)$ (i.e., the initial state belongs to some macrostate). In that case, $j \in \mathrm{dom}(g)$ by Condition 3 of Definition 8. In addition, j is clearly always within $\mathrm{img}(\gamma)$. Thus, when $i \in \mathrm{dom}(g)$, $j \in D$ (by Eq. (2)) and $j' \in \mathrm{img}(\omega)$ (by Eq. (3)).

Finally, define $\mathcal{X} := \mathrm{img}(\omega)$ (i.e., the set of "canonical" microstates). By definition of ω, $\mathcal{X} \subseteq \mathrm{dom}(g)$ (and therefore also $\mathcal{X} \subseteq \mathcal{Y}$). Note also that $|\mathcal{Z}| = |\mathcal{X}|$, since ω is one-to-one. Consider the restriction of W to $\mathcal{X}$, which we indicate by $W^{\mathcal{X}}$. Since W is a product of 0/1 valued stochastic matrices, both W and its restriction $W^{\mathcal{X}}$ are 0/1 valued. Furthermore, for any input state $i \in \mathcal{X}$, $i \in \mathrm{dom}(g)$; therefore, by Condition (b) in the above list, the j that satisfies $W_{ji} = 1$ itself obeys $j \in \mathcal{X}$. Combining these results with Condition 3 of Definition 8 states that $W^{\mathcal{X}}$ is a valid stochastic matrix that carries out

$$W^{\mathcal{X}}_{ji} = \delta(j, \omega(\hat{f}(g(i)))) = \delta(j, \omega(\hat{f}(\omega^{-1}(i)))) \,, \tag{5}$$

where we've used the fact that $\omega^{-1} = g$ over $\mathcal{X}$.

Supplementary Note 6: Restricted set of idempotents

To illustrate some of the issues a restriction on which idempotents can be implemented raises, consider the case where our full system is a set of N visible spins plus an unspecified set of hidden spins. Suppose the only idempotent functions we can apply to our system are those that affect either one or two spins at a time, leaving all the others unchanged. Physically, this would mean that the Hamiltonian of our system is a sum of one-spin and two-spin terms. (We then implement an idempotent function by dynamically altering the relative strengths of those terms.)

We can implement any function over the set of N spins using this set of idempotent functions if the set of hidden spins is large enough — so long as the idempotent functions allow us to change any set of one or two spins. (The analysis if we can only change pairs of spins that are neighbors on a lattice, as in an Ising spin, is more complicated.) To see this, first note that we can use such an idempotent function to copy the state of a spin into a different "target" spin. By repeating this function with different target spins, we can make any desired number of copies of the original spin. Next, note that another of our allowed idempotent functions maps any spin-pair $(x_1, x_2) \to (0, \mathsf{NAND}(x_1, x_2))$, i.e., evaluates the NAND of the two spins and stores the result in the second spin. So if we make a copy of both x_1 and x_2, and then run this NAND idempotent function on that pair of copy-bits, we will have implemented a full NAND gate whose input bits were x_1 and x_2 and whose output bit is $\mathsf{NAND}(x_1, x_2)$. (We will also have zeroed the copy-bit that doesn't equal $\mathsf{NAND}(x_1, x_2)$,

but that doesn't matter.)

Now NAND is a universal logical gate, meaning that we can implement any Boolean function $f : \{0,1\}^N \rightarrow \{0,1\}^N$ by appropriately connecting NAND gates [5] into one another. (In general, such an implementation will require that some of the gates have fanout greater than 1 — but we can implement an arbitrary fanout, by repeated using our bit-copy idempotent function.) So by using enough hidden states and an appropriate set of two-spin idempotent functions, we can evaluate the (arbitrary) function f of the N visible spins, storing the resultant output in N of the hidden spins. At that point we can copy the (hidden) output back to the (visible) input bits, thereby completing the process of running f on those input bits.

In general, implementing f with this construction will require more hidden states and more hidden timesteps than would implementing it using arbitrary idempotent functions. However, calculating the associated increase in the space and time costs can be quite challenging. The time cost in our construction is given by the depth of the circuit of NAND gates and the fanouts of those gates. On the other hand, the number of hidden states is determined by the number and type of gates in that circuit. The analysis of how these quantities and their tradeoff depends on the function f is closely related to ongoing research in circuit complexity theory [6, 7].

Moreover, there seems to be no reason to believe that using our set of allowed idempotent functions to make circuits of NAND gates is the most efficient way to use them. In general there will be a complicated tradeoff between re-using hidden spins to implement multiple gates (thereby reducing the total number of hidden spins needed) and increasing the number of gates that can be operated in parallel (which reduces the total number of timesteps).

SUPPLEMENTARY REFERENCES

[1] Giovanni Diana, G Baris Bagci, and Massimiliano Esposito. Finite-time erasing of information stored in fermionic bits. *Physical Review E*, 87(1):012111, 2013.

[2] Massimiliano Esposito. Stochastic thermodynamics under coarse graining. *Physical Review E*, 85(4):041125, 2012.

[3] Massimiliano Esposito and Christian Van den Broeck. Three faces of the second law. i. master equation formulation. *Physical Review E*, 82(1):011143, 2010.

[4] Jeremy A Owen, Artemy Kolchinsky, and David H Wolpert. Number of hidden states needed to physically implement a given conditional distribution. *New Journal of Physics*, 21(1):013022, 2019.

[5] M Morris Mano, Charles R Kime, Tom Martin, et al. *Logic and computer design fundamentals*, volume 3. Prentice Hall, 2008.

[6] Sanjeev Arora and Boaz Barak. *Computational complexity: a modern approach*. Cambridge University Press, 2009.

[7] John E Savage. *Models of computation*, volume 136. Addison-Wesley Reading, MA, 1998.
